# COVID-19 Pandemic and the Developmental Health of Kindergarteners

**DOI:** 10.1001/jamapediatrics.2024.7057

**Published:** 2025-03-10

**Authors:** Judith L. Perrigo, Jordan Morales, Nicholas Jackson, Magdalena Janus, Lisa Stanley, Mitchell Wong, Neal Halfon

**Affiliations:** 1 Department of Social Welfare, University of California, Los Angeles (UCLA) Luskin School of Public Affairs; 2 UCLA Center for Healthier Children, Families and Communities, Los Angeles, California; 3 Department of Medicine Statistics Core, University of California, Los Angeles; 4 Offord Centre for Child Studies, Department of Psychiatry and Behavioural Neurosciences, McMaster University, Hamilton, Ontario, Canada; 5 Department of Medicine, David Geffen School of Medicine at University of California, Los Angeles; 6 Department of Pediatrics, David Geffen School of Medicine, University of California, Los Angeles; 7 Department of Health Policy and Management, Fielding School of Public Health, University of California, Los Angeles

## Abstract

**Importance:**

Recent studies have associated the COVID-19 pandemic with negative developmental outcomes in children. However, research focused on young children remains limited, with few studies including multiple years of pre- and postpandemic onset data.

**Objective:**

To examine the impact of the COVID-19 pandemic on US kindergarteners’ developmental health.

**Design, Setting, and Participants:**

This repeated cross-sectional panel study examined developmental health trends, as measured by the Early Development Instrument (EDI), among a convenience sample US kindergarteners from 2010 to 2023. EDI data were obtained from 390 school districts across 19 states. Data were analyzed from June December 2024.

**Exposure:**

Kindergarteners’ developmental health was compared between prepandemic (2018 to 2020) and postpandemic (2021 to 2023) onset cohorts.

**Main Outcomes and Measures:**

Outcomes were EDI scores across time in 5 domains: (1) physical health and well-being, (2) social competence, (3) emotional maturity, (4) language and cognitive development, and (5) communication and general knowledge. The mean (95% CI) EDI scores were assessed.

**Results:**

In this sample of of 475 740 US kindergarten students, 242 869 were male (51.1%), there were 53 841 African American or Black students (11.4%), 263 037 Hispanic or Latino/a students (55.5%), and 95 258 White students (20.1%), and the mean (SD) age was 6 (0.4) years (range, 4.0-8.0 years). Compared with the immediate prepandemic onset period, the rate of change in EDI scores was significantly lower following the pandemic onset in language and cognitive development (mean change, −0.45; 95% CI, −0.48 to −0.43), social competence (mean change, −0.03; 95% CI, −0.06 to −0.01), and communication and general knowledge (mean change, −0.18; 95% CI, −0.22 to −0.15). EDI scores were significantly higher in emotional maturity (mean change, 0.05; 95% CI, 0.03 to 0.07), and no significant changes were observed in the physical health and well-being domain (mean change, 0; 95% CI, −0.01 to 0.02).

**Conclusions and Relevance:**

The COVID-19 pandemic was associated with varying developmental health outcomes in kindergarteners. Negative developmental trends existed immediately before the pandemic, with most persisting or slowing postpandemic onset. These results highlight troubling trends in kindergarteners’ development, both before and during the pandemic, and more information is needed to understand why developmental outcomes are worsening over time.

## Key Points

**Question:**

How did the COVID-19 pandemic affect US kindergarteners’ developmental health?

**Findings:**

In this cross-sectional study of 475 740 kindergarten students, no changes were found in physical health and well-being and significant increases in emotional maturity. Significant decreases were observed in language and cognitive development, social competence, and communication and general knowledge.

**Meaning:**

These findings suggest that many negative trends observed in kindergarteners’ developmental health during the COVID-19 pandemic were small and existed prior to its onset.

## Teaser

This cross-sectional study examines the developmental health of US kindergarteners before and after the COVID-19 pandemic using data from the Early Development Instrument.

## Introduction

Disruptions in a child’s developmental ecosystem, such as the COVID-19 pandemic, can affect their physical, socioemotional, and cognitive development.^1^ For school-age children, the pandemic has been linked to worse academic and behavioral health outcomes. US academic standardized test scores for third to eighth graders were worse in school districts that had longer lockdowns and offered less in-person instruction, and this association was particularly pronounced among students from minoritized racial and ethnic groups.^2,3,4,5,6,7,8,9,10^ Schools are not only a place of learning but also where children develop social skills and build relationships.^11^ Thus, school closures reduced children’s opportunities for socialization.^12^

Before the COVID-19 pandemic, children (aged 0 to 17 years) had declining health and well-being regarding anxiety, depression, and physical activity.^13^ Since the pandemic, some trends have worsened (eg, lower levels of physical activity) and new concerning trends have emerged (eg, behavior or conduct problems).^13^ Prior studies routinely included only a few months or years of pre- and postpandemic onset data.^13^ This limited their ability to clearly determine long-standing or ongoing population needs. Longitudinal studies with extensive pre- and postpandemic onset data are necessary to capture potential trends that existed long before the pandemic, those that continue to persist years after its onset, and those that have emerged for the first time. Also, many COVID-19–related child studies have covered a wide age range from 0 to 18 years,^13,14,15,16^ neglecting the fact that children have distinct needs at different developmental phases. During early childhood, children are especially vulnerable to significant disruptions like pandemics given their increased sensitivity to adversity, emerging coping skills, and substantial reliance on caregivers.^17,18,19,20^ Focusing on early childhood is also optimal because findings can provide insights into prevention and early intervention initiatives, potentially benefiting children at all ages. Specifically, early intervention has the potential to significantly affect not only immediate developmental health needs, such as developmental delays or behavioral challenges, but also those in middle childhood, adolescence, and beyond.^21,22^

Equitable investments in kindergarteners can lead to better health outcomes across the life course.^23,24^ Thus, the primary goal of the current study was to examine the association of the COVID-19 pandemic with the early childhood developmental health trends of US kindergarteners before (2010 to 2020) and after (2021 to 2023) the pandemic onset. We used the Early Development Instrument (EDI), an internationally validated, population-level measurement of holistic developmental health and school readiness in kindergarten^25,26^ that has been routinely implemented in several US regions. Using 14 years of EDI data, this study aimed to identify longitudinal trends (2010 to 2023) before and during the pandemic and compare early childhood developmental health trends immediately before (2018 to 2020) and after (2021 to 2023) the pandemic onset. We hypothesized that EDI scores would differ from the immediate prepandemic trend and decrease across all 5 developmental domains following the COVID-19 pandemic onset.

## Method

### Design, Study Setting, and Sample

This repeated cross-sectional study followed the Strengthening the Reporting of Observational Studies in Epidemiology (STROBE) reporting guideline. All procedures regarding data collection, analysis, and reporting were approved by the University of California Los Angeles (UCLA) institutional review board.

EDI data are primarily collected to support local communities identify optimal resource allocation, which include addressing concerns associated with population-level child development. Due to the pragmatic nature of this data collection, the sampling strategy varies from site to site. Some sites only collect data for 1 year, whereas others collect data multiple times over several years. EDI data were collected in various communities between 2010 and 2023. During this period, 548 537 kindergarteners were surveyed in 19 states and 398 school districts across the US. Students who had at least 1 completed EDI domain were included in the analysis.

### Measurement

There is ample evidence of the EDI’s robust psychometric properties from the US, Canada, Australia, and other countries.^25,27,28,29^ Kindergarten teachers report on each student in their class across 5 domains: (1) physical health and well-being (13 items), (2) social competence (26 items), (3) emotional maturity (30 items), (4) language and cognitive development (26 items), and (5) communication and general knowledge (8 items). The EDI also includes demographic information, such as age, sex at birth, and ethnoracial background. Given the existing literature on the varied impacts of the COVID-19 pandemic across minoritized racial and ethnic groups,^30^ race and ethnicity were determined an important variable to include in the analysis. Race and ethnicity were parent-reported to the school and captured in EDI data. Covariates included ethnoracial background, sex, and the National Neighborhood Equity Index, which is a measure of neighborhood socioeconomic context.^31^

### Data Collection

All US EDI data are maintained at UCLA. Caregivers received an informational letter containing an opt-out option. With passive caregiver consent, teachers complete the EDI at least 3 months into the school year to ensure familiarity with their students.

### Statistical Analysis

**Figure 1.**
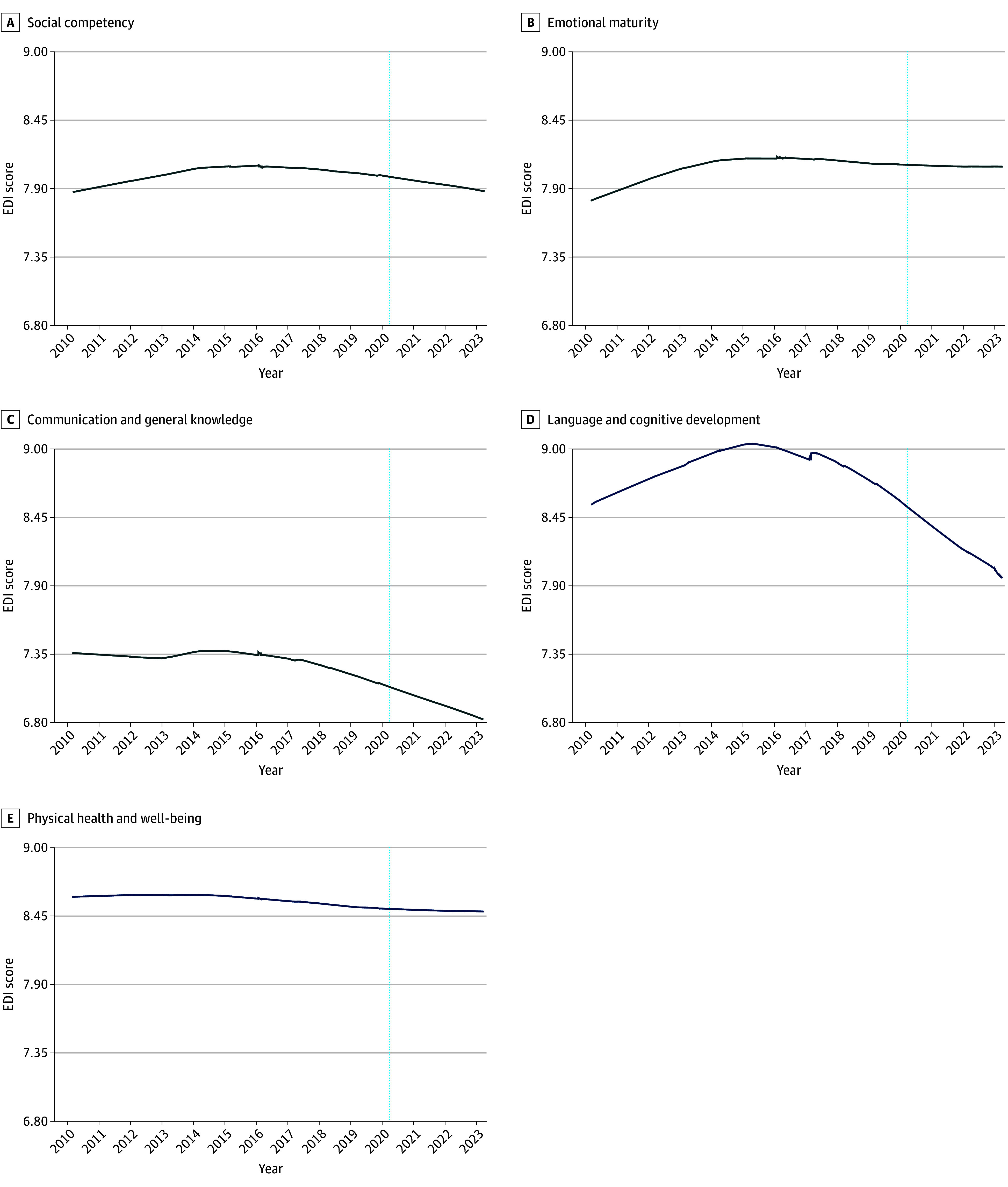
Locally Estimated Scatterplot Smoothing Plots Illustrate Longitudinal Trends (2010-2023) in Mean Scores for 5 Early Development Instrument (EDI) Domains The vertical blue lines mark the onset of the COVID-19 pandemic.

Locally estimated scatterplot smoothing plots were used to visually examine how the 5 EDI domains changed from 2010 through 2023 (Figure 1). Four distinct periods were identified based on observations that scores were (1) stable or increasing (2010 to 2015), (2) stable or decreasing (2016 to 2017), and (3) decreasing (2018 to 2020), and (4) to test for postpandemic onset changes (2021 to 2023).

Interrupted time-series modeling was used to estimate 2 models examining (1) mean EDI scores between periods and (2) yearly linear rate-of-change differences across periods. These models accounted for repeated measures over time in schools and time-invariant confounding between schools using school fixed effects. Our models for mean EDI scores included a fixed effect categorical variable for time-period and our models examining rate of change differences additionally incorporated a linear term for continuous time with a time-by-time period interaction term. A sensitivity analysis was conducted that restricted schools with at least 2 EDI measurements in the immediate pre- and postpandemic onset periods (eTable 1 in Supplement 1). Missingness was also assessed (eTable 2 in Supplement 1). All analyses were conducted in Stata MP version 18. Statistical significance was set at *P* < .05, and all tests were 2-sided. Data were analyzed from June to December 2024.

## Results

The EDI was collected for 548 537 kindergarteners in 19 states and 398 school districts between 2010 and 2023. Of those, 475 740 (86.7%) had at least 1 complete EDI domain scored. These observations were retained for analysis, representing 3630 schools, 390 school districts, and 19 states.

The sample consisted of 242 869 male participants (51.1%) and 232 612 female participants (48.9%). The study included 53 841 African American or Black students (11.4%), 34 282 Asian American, Hawaiian, or Pacific Islander students (7.2%), 263 037 Hispanic or Latino/a students (55.5%), 95 258 White students (20.1%), and 27 603 students from other ethnoracial backgrounds (including American Indian, Alaska Native, and all other categories of races and ethnicities) (5.8%). Most of the sample came from California (183 827 students [38.6%]) and Texas (184 596 [38.8%]). The mean (SD) age was 6.0 (0.4) years (range, 4.0-8.0 years). Demographic characteristics and state data across 4 periods (ie, 3 prepandemic onset periods and 1 postpandemic onset period) can be found in Table 1.

**Table 1.** Frequency Distribution of US Kindergarteners’ Descriptive Early Development Instrument Data, 2010 to 2023 (n = 475 740)

Characteristic	Participants, No. (%)				
	Total	2010-2015	2016-2017	2018-2020 (Immediate prepandemic onset)	2021-2023 (Postpandemic onset)
Study sample	475 740 (100)	194 820 (41.0)	80 734 (17.0)	114 359 (24.0)	85 827 (18.0)
Schools, No.	3630	1690	932	1469	1233
Years a school was surveyed, mean (SD) [range]	1.8 (1.7) [1-13]	2.3 (1.8) [1-11]	1.9 (1.2) [1-7]	1.8 (1.1) [1-8]	2.0 (0.9) [1-6]
Students per school, median (IQR)	83 (54-143)	91 (61-138)	75 (52-108)	70 (48-95)	62 (43-86)
Sex					
Male	242 869 (51.1)	100 153 (51.4)	41 216 (51.1)	57 914 (50.6)	43 586 (50.8)
Female	232 612 (48.9)	94 573 (58.6)	39 378 (48.9)	56 440 (49.4)	42 221 (49.2)
Race and ethnicity					
African American or Black	53 841 (11.4)	26 028 (13.4)	10 233 (19.0)	10 783 (9.4)	6757 (7.9)
Asian American, Native Hawaiian, or Pacific Islander	34 282 (7.2)	9619 (5.0)	5391 (6.7)	10 808 (9.5)	8464 (9.9)
Hispanic or Latino/a	263 037 (55.5)	112 808 (58.1)	46 669 (57.9)	61 520 (53.9)	42 040 (49.3)
Other^a^	27 603 (5.8)	9914 (5.1)	2996 (3.7)	7719 (6.8)	6974 (8.2)
White	95 258 (20.1)	35 672 (18.4)	15 245 (18.9)	23 354 (20.5)	20 987 (20.1)
State					
Arkansas	6511 (1.4)	NA	NA	2734 (2.4)	3777 (4.4)
California	183 827 (38.6)	61 920 (31.8)	26 492 (32.8)	54 746 (47.9)	40 669 (47.4)
Connecticut	4523 (1.0)	2395 (1.2)	585 (0.7)	1386 (1.2)	157 (0.2)
District of Columbia^b^	10 804 (2.3)	1780 (0.9)	4602 (5.7)	3878 (3.4)	544 (0.6)
Florida	20 032 (4.2)	13 857 (7.1)	6175 (7.6)	NA	NA
Illinois	3300 (0.7)	NA	1127 (1.4)	NA	2173 (2.5)
Kansas	556 (0.1)	556 (0.3)	NA	NA	NA
Louisiana	5872 (1.2)	5872 (3.0)	NA	NA	NA
Michigan	7369 (1.5)	7369 (3.8)	NA	NA	NA
Mississippi	3072 (0.6)	3072 (1.6)	NA	NA	NA
New York	2398 (0.5)	1834 (0.9)	334 (0.4)	230 (0.2)	NA
North Carolina	1082 (0.2)	NA	NA	1082 (0.9)	NA
Ohio	656 (0.1)	656 (0.3)	NA	NA	NA
Oklahoma	7124 (1.5)	7124 (3.7)	NA	NA	NA
South Carolina	8957 (1.9)	NA	2944 (3.6)	1961 (1.7)	4052 (4.7)
Tennessee	1770 (0.4)	562 (0.3)	NA	1208 (1.1)	NA
Texas	184 596 (38.8)	86 948 (44.6)	38 475 (47.7)	36 420 (31.8)	22 753 (26.5)
Virginia	22 416 (4.7)	NA	NA	10 714 (9.4)	11 702 (13.6)
Washington	875 (0.2)	875 (0.4)	NA	NA	NA

Abbreviation: NA, not available.

^a^ Other includes multiracial, American Indian, Alaska Native, and other ethnoracial categories that were not identified.

^b^ Although Washington, DC, is not a state, we included it in the analysis.

### School EDI Mean Scores Across Periods

**Figure 2.**
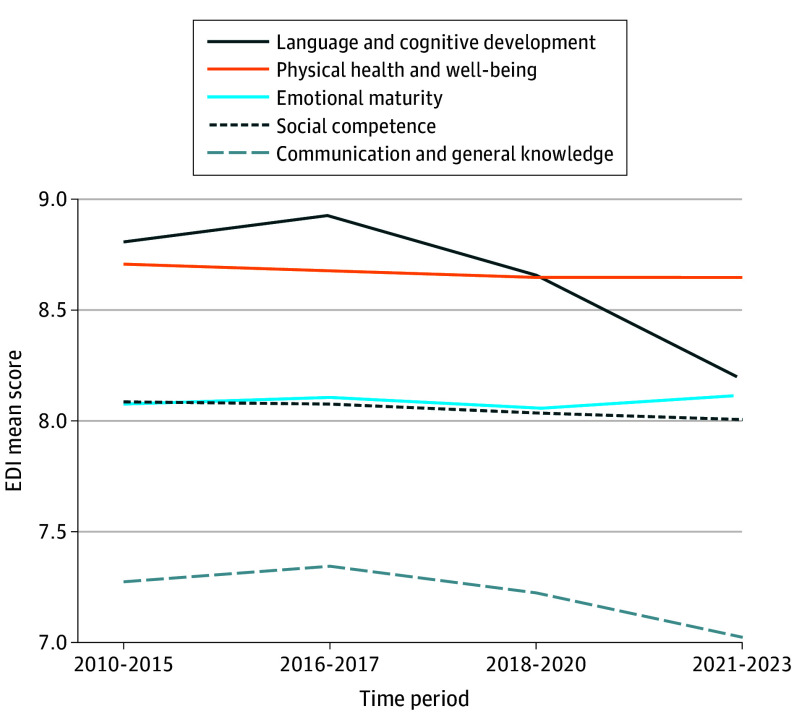
Trends in Mean Early Development Instrument (EDI) Scores Are Presented Across 4 Time Periods: 2010-2015, 2016-2017, 2018-2020, and 2021-2023

Children’s mean scores on the 5 EDI domains, aggregated at the school level and representing 3630 schools, were examined across the 4 periods (Figure 2). Compared with the immediate prepandemic onset period (2018 to 2020), mean EDI scores were significantly lower during the pandemic onset in language and cognitive development, social competence, and communication skills and general knowledge domains; higher in emotional maturity; and unchanged in physical health and well-being (eTable 1 in Supplement 1).

Differences in EDI domains mean scores among all 4 periods were also tested (Table 2). The language and cognitive development domain scores showed the greatest decline between earlier time periods and the immediate prepandemic onset period (2018 to 2020), with mean differences of −0.15 (95% CI, −0.17 to −0.13) when compared with the 2010 to 2015 period and −0.27 (95% CI, −0.29 to −0.25) when compared with the 2016 to 2017 period.

**Table 2.** Differences in Early Development Instrument Domains Mean Scores by Time Period, 2010 to 2023 (3360 Schools)

EDI domain	Time period, mean (95% CI)				Comparison, Δ mean (95% CI)		
	1: 2010-2015	2: 2016-2017	3: 2018-2020	4: 2021-2023	1 vs 3	2 vs 3	4 vs 3
Language and cognitive development	8.81 (8.80 to 8.82)	8.93 (8.91 to 8.94)	8.66 (8.65 to 8.67)	8.19 (8.17 to 8.21)	0.15 (0.13 to 0.17)	0.27 (0.25 to 0.29)	−0.47 (−0.49 to −0.45)
Social competence	8.93 (8.91 to 8.94)	8.08 (8.06 to 8.10)	8.04 (8.03 to 8.06)	8.01 (7.99 to 8.03)	0.05 (0.03 to 0.07)	0.04 (0.01 to 0.06)	−0.03 (−0.06 to 0)
Emotional maturity	8.08 (8.07 to 8.09)	8.11 (8.09 to 8.12)	8.06 (8.05 to 8.07)	8.12 (8.10 to 8.14)	0.02 (0 to 0.04)	0.04 (0.02 to 0.06)	0.06 (0.03 to 0.08)
Communication and general knowledge	7.28 (7.26 to 7.30)	7.35 (7.33 to 7.37)	7.23 (7.21 to 7.25)	7.03 (7.00 to 7.06)	0.05 (0.02 to 0.08)	0.12 (0.09 to 0.15)	−0.21 (−0.24 to −0.17)
Physical health and well-being	8.71 (8.70 to 8.72)	8.68 (8.67 to 8.69)	8.65 (8.64 to 8.66)	8.65 (8.64 to 8.66)	0.06 (0.04 to 0.07)	0.03 (0.01 to 0.05)	0 (−0.02 to 0.02)

Abbreviation: EDI, Early Development Instrument.

The lowest EDI scores in 3 domains—communication and general knowledge, language and cognitive development, and social competence—were observed in the postpandemic onset period (2021 to 2023). These scores were significantly lower compared with the immediate prepandemic onset period (2018 to 2020) for communication and general knowledge (mean change, −0.21; −0.24 to −0.17), language and cognitive development (mean change, −0.47; −0.49 to −0.45), and social competence (mean change, −0.03; −0.06 to −0.01). There was no difference between the immediate pre- and postpandemic onset periods in physical health (mean change, 0). In contrast, the postpandemic onset cohort showed higher scores in the emotional maturity domain compared with the immediate prepandemic onset period (mean change, 0.06). Sensitivity analyses showed similar results in models with at least 2 measurements per period (eTable 1 in Supplement 1).

### Rate of Year EDI Score Change Across Periods

Linear slopes for change in EDI scores were estimated in each period and compared across periods relative to the immediate prepandemic onset (2018 to 2020). This rate of decreasing EDI scores was significantly greater than the rate of change from the 2010 to 2015 period across all domains (Table 3). The rate decreased significantly faster than during the 2016 to 2017 period for communication and general knowledge (mean change, −0.13; 95% CI, 0.10 to 0.16) and language and cognitive development domains (mean change, −0.28; 0.26 to 0.30). In the postpandemic onset period, the only domain with an observed faster rate of decline in EDI score compared with the prepandemic onset period was emotional maturity (mean change, −0.05; 95% CI, 0.03 to 0.07). Although largely still decreasing, rates of change in the postpandemic onset period for communication and general knowledge (mean change, 0.18; 95% CI, −0.22 to −0.15), language and cognitive development (mean change, 0.45; −0.48 to −0.43), and physical health (mean change, 0; −0.02 to 0.02) declined at a rate slower than in the prepandemic onset period. For the social competence domain, no reliable difference in the rate of change occurred relative to the prepandemic onset period. Sensitivity analyses showed similar results for models with at least 2 measurements per period (eTable 1 in Supplement 1)

**Table 3.** Fixed-Effects Mean Scores and Effect Size Adjusted for Ethnoracial Background, Sex, and National Neighborhood Equity Index, 2010-2023 (3515 Schools)

EDI domain	Time period				Comparison of time periods		
	1: 2010-2015	2: 2016-2017	3: 2018-2020	4: 2021-2023	1 vs 3	2 vs 3	4 vs 3
**Mean (95% CI)**							
Language and cognitive development	8.81 (8.80 to 8.83)	8.95 (8.93 to 8.96)	8.67 (8.66 to 8.68)	8.22 (8.20 to 8.23)	0.15 (0.13 to 0.17)	0.28 (0.26 to 0.30)	−0.45 (−0.48 to −0.43)
Social competence	8.11 (8.10 to 8.12)	8.09 (8.07 to 8.11)	8.05 (8.04 to 8.06)	8.02 (8.00 to 8.04)	0.06 (0.04 to 0.08)	0.04 (0.02 to 0.07)	−0.03 (−0.06 to −0.01)
Emotional maturity	8.10 (8.09 to 8.11)	8.11 (8.10 to 8.13)	8.07 (8.06 to 8.08)	8.12 (8.10 to 8.14)	0.03 (0.01 to 0.04)	0.05 (0.03 to 0.06)	0.05 (0.03 to 0.07)
Communication and general knowledge	7.28 (7.26 to 7.29)	7.36 (7.34 to 7.38)	7.23 (7.21 to 7.25)	7.05 (7.02 to 7.08)	0.04 (0.02 to 0.07)	0.13 (0.10 to 0.16)	−0.18 (−0.22 to −0.15)
Physical health and well-being	8.71 (8.70 to 8.72)	8.68 (8.66 to 8.69)	8.65 (8.64 to 8.66)	8.65 (8.63 to 8.66)	0.06 (0.05 to 0.08)	0.03 (0.01 to 0.05)	0 (−0.01 to 0.02)
**Cohen *d* (95% CI)**							
Language and cognitive development	0.07 (0.07 to 0.08)	0.15 (0.14 to 0.16)	−0.02 (−0.02 to −0.01)	−0.27 (−0.28 to −0.26)	0.09 (0.08 to 0.10)	0.16 (0.15 to 0.17)	−0.25 (−0.26 to −0.24)
Social competence	0.02 (0.01 to 0.02)	0.01 (0 to 0.02)	−0.01 (−0.02 to 0)	−0.03 (−0.04 to −0.02)	0.03 (0.02 to 0.04)	0.02 (0.03 to 0.01)	−0.02 (−0.03 to −0.01)
Emotional maturity	0 (−0.01 to 0.01)	0.01 (0 to 0.02)	−0.02 (−0.02 to −0.01)	0.01 (0 to 0.02)	0.02 (0.01 to 0.03)	0.03 (0.02 to 0.04)	0.03 (0.02 to 0.04)
Communication and general knowledge	0.02 (0.01 to 0.02)	0.05 (0.04 to 0.05)	0 (−0.01 to 0)	−0.07 (−0.08 to −0.06)	0.02 (0.01 to 0.03)	0.05 (0.04 to 0.06)	−0.07 (−0.08 to −0.06)
Physical health and well-being	0.02 (0.02 to 0.03)	0 (−0.01 to 0.01)	−0.02 (−0.03 to −0.01)	−0.02 (−0.03 to −0.01)	0.04 (0.03 to 0.05)	0.02 (0.01 to 0.03)	0 (−0.02 to 0.01)

Abbreviation: EDI, Early Development Instrument.

## Discussion

This repeated cross-sectional study found significant changes in kindergarteners’ developmental health before and during the COVID-19 pandemic. Although we hypothesized that the pandemic would decrease across all 5 EDI domains, our results suggested a more complex picture. In a sample of approximately half a million children from 19 states across 14 years, the direction and rate of kindergarteners’ developmental trends varied across domains. Compared with the immediate prepandemic onset cohort (2018 to 2020), mean EDI scores in the postpandemic onset cohort (2021 to 2023) were the lowest in language and cognitive development, social competence, and communication and general knowledge, whereas scores were highest in emotional maturity and did not differ for physical health and well-being. The domains of language and cognitive development and communication and general knowledge were most severely affected, the likely result of school closures and virtual learning environments necessitated by COVID-19 public health measures.^32^ These measures also limited children’s usual social opportunities with peers and adults who are not caregivers, which may explain the observed slight decrease in social competence. The increased exposure to adult stressors during the pandemic (eg, COVID-19 casualty counts, financial strain, health anxiety, news coverage)^33^ may also account for the increase in emotional maturity detected in our sample. Increased maturity may have been required for young children to cope with pandemic-related stressors, similar to maturation observed in children facing significant challenges, such as family separation or poverty.^34,35^ However, these observed changes in mean scores for emotional maturity and social competence between pre- and postpandemic onset cohorts are slight and may be an artifact of the study’s large sample size.

The extended length of our 14-year study period also revealed that EDI scores were already significantly decreasing across all domains in the immediate prepandemic period (2018 to 2020). This finding supports literature highlighting concerning national trends in the health and well-being of children,^13,36,37^ attributed to various factors, including excessive screen time,^38^ economic instability,^39^ and climate change.^40^ Although early childhood education and care interventions aim to reverse these trends, their effectiveness is hindered by inequitable access, funding challenges, and increased financial strain on families.^41,42,43,44^ These negative trends occurred at the same or slower rate during the postpandemic onset period (2021 to 2023), except for emotional maturity, which decreased at a faster than the immediate prepandemic onset period. The initial increase in emotional maturity observed from the pre- to postpandemic onset periods now seems to be reversing. With children returning to school and parents resuming in-person work, it is likely that more recent kindergarten cohorts are once again being shielded from adult stressors, possibly contributing to the rapid decline in emotional maturity scores. The slowing of these negative trends across all 5 EDI domains in the postpandemic onset period may also be attributed to several pandemic-related federal measures aimed at supporting children and caregivers, such as stimulus checks, tax credits, the Tenant Relief Act, and expanded unemployment insurance benefits, which collectively reduced the child poverty rate by 76% in 2021.^45^ Researchers should continue to track kindergarten cohorts to assess how trends evolve as pandemic-related supports are phased out.

The children in our postpandemic onset cohort experienced pandemic-related restrictions as preschoolers, which likely limited opportunities for play and social interactions with peers and extended family members but increased interactions with caregivers. Language and cognitive development saw the largest decline between pre- and postpandemic onset cohorts, likely reflecting reduced play, in-person interactions, and early learning opportunities due to early childhood center closures. Consistent with our findings, other smaller-scale investigations on the association between the COVID-19 pandemic and early childhood show overall negative impacts on language conceptualization and cognitive development.^46,47^ Yet differential pandemic-related impacts in language and cognitive development have been observed, including less severe developmental losses and increased vocabulary and cognitive performance in young children from higher socioeconomic circumstances.^47,48^ Given that our large study sample reflects multiple geographic regions and ethnoracial backgrounds, our results may be more generalizable and inclusive of the developmental trajectories of a diverse range of children, including those from lower socioeconomic statuses. This is particularly important given the disproportionately negative impacts of COVID-19 on families who are socioeconomically disadvantaged and from minoritized racial and ethnic groups.^49,50^ Additionally, our findings contradict studies that showed declines in young children’s socioemotional development related to COVID-19,^51,52^ which may reflect our ability to analyze trends over 14 years, rather than only during pre- and postpandemic onset. Moreover, the EDI measured 2 related but distinct aspects of socioemotional development separately (social competence and emotional maturity), which may have allowed for greater nuance in our results.

Considering the current study’s relevance to public health policy, it is important to recall the unique aspect of the EDI’s conceptual and design background which has been its feasibility for use with large populations.^53^ The population-wide design has 2 advantages. First, including all children in a given region enhances the community ownership of the results, and second, it makes the EDI suitable for identifying broad trends that might have important policy implications.^54^ For example, a study in Gold Coast, Australia, found that there were fewer clinicians of speech and language services in areas where children’s language and communication domains showed higher vulnerability rates, and most of those available were private, thus more costly.^55^ A Canada-wide examination of teacher reports of anxious behaviors within 1 of the EDI domains between 2004 and 2015 showed changes between years and jurisdictions, however, these were small in size.^56^ Availability of population-wide early child development data from early decades of the 21st century is increasingly becoming useful in the countries using the EDI to examine the potential trend changes in the postpandemic years.^57^

### Study Limitations

Our study has several limitations. Because EDI data are collected for programmatic purposes, this study was limited by the necessary grouping of data into pre- and postpandemic onset cohorts of differing durations (ie, 2010 to 2015, 2016 to 2017, 2018 to 2020, and 2021 to 2023). Also, EDI data are reported by teachers and capture children’s developmental abilities and behaviors in school environments, which may differ from those observed at home. Yet, links between children’s behavior in the classroom and at home have been observed through preschool-based interventions,^58,59^ so this discrepancy may be slight. Trends in preschool attendance and its association with developmental outcomes could not be examined due to a lack of reliable preschool attendance data in this study. Our study was also limited by a lack of availability on individual students’ socioeconomic status, preventing insight into how it influenced the association between the COVID-19 pandemic and EDI scores. However, our models included the National Neighborhood Equity Index^31^ to control for neighborhood socioeconomic context, which didn’t have meaningful differences.

Although we detected significant differences in mean EDI scores and their rate of change over time, the reliably small effect sizes suggest limited practical meaning. However, EDI scores have been linked to tangible outcomes, such as academic proficiency.^27,60^ Future research should investigate whether these small effect sizes have meaningful public health and education implications. Finally, the composition of states contributing data to each study time period varied, limiting the generalizability of our findings. However, the data predominantly comes from California and Texas, 2 states with distinct pandemic policies for kindergarteners (eg, in person vs virtual classrooms). Efforts to increase the use of the EDI nationally should be pursued to increase the generalizability of future studies using this measure.

## Conclusions

Kindergarteners’ developmental health is an indicator of early childhood (birth to age 5 years) outcomes and a factor of future health trajectories.^61^ Children’s developmental health is also very susceptible to environmental influences, such as school disruptions during the COVID-19 pandemic. Our results identify public health prevention domains for kindergarten populations with important implications for school and health policies as the pandemic persists. The negative developmental trends observed before the pandemic highlight broader challenges in early childhood development in the US, including the association of growing wealth inequality, challenges in strengthening the early childhood workforce, and disparities in access to care and education. Our findings underscore the need for early childhood policies that address these preexisting challenges and the additional stressors introduced by the pandemic.

Our results could apply to future pandemics or collective crises (a time of acute difficulty) that force children out of school, such as mass shooting events, terrorist attacks, and natural disasters. The initial and ongoing responses of leaders to crises can offer lessons for the future, which is particularly important for vulnerable populations (eg, low socioeconomic status) with fewer resources to buffer against potential hardships.^62^

## Notes

Supplement 1. **eTable 1.** Sensitivity Analysis of Fixed-Effects Mean Scores and Rates of Change Per Year: At Least 2 Measures, 2010 to 2023 (105 Schools; 76 765 Students) **eTable 2.** Distribution of Missingness: Sex, Race and Ethnicity, and State, 2010 to 2023

Supplement 2.
Data Sharing Statement
