# Supplementary material for: COVID-19 Pandemic and the Developmental Health of Kindergarteners
Source: JAMA Pediatr. 2025 Mar 10;179(5):550–8. doi: 10.1001/jamapediatrics.2024.7057 (PMC11894545; doi:10.1001/jamapediatrics.2024.7057)
Supplement: Supplement 1. — eTable 1. Sensitivity Analysis of Fixed-Effects Mean Scores and Rates of Change Per Year: At Least 2 Measures, 2010 to 2023 (105 Schools; 76 765 Students) eTable 2. Distribution of Missingness: Sex, Race and Ethnicity, and State, 2010 to 2023 [file jamapediatr-e247057-s001.pdf]

Supplemental Online Content

Perrigo JL, Morales J, Jackson N, et al. COVID-19 Pandemic's Impact on US Kindergarteners. *JAMA Pediatr*. Published online March 10, 2025. doi:10.1001/jamapediatrics.2024.7057

eTable 1. Sensitivity Analysis of Fixed-Effects Mean Scores and Rates of Change Per Year: At Least 2 Measures, 2010 to 2023 (105 Schools; 76 765 Students)

eTable 2. Distribution of Missingness: Sex, Race and Ethnicity, and State, 2010 to 2023

This supplemental material has been provided by the authors to give readers additional information about their work.

© 2025 Perrigo JL et al. *JAMA Pediatr*.

eTable 1. Sensitivity Analysis of Fixed-Effects Mean Scores and Rates of Change Per Year: At Least 2 Measures, 2010 to 2023 (105 Schools, 76 765 Students)

EDI Domain	Time Period, *M*(95% CI)				Comparison Δ*M*(95% CI)		
	1: 2010–2015	2: 2016–2017	3: 2018–2020	4: 2021–2023	1 vs. 3	2 vs. 3	4 vs. 3
Language & Cognitive Development	9.11 (9.09, 9.13)	9.22 (9.19, 9.24)	8.84 (8.81, 8.86)	8.18 (8.15, 8.21)	0.28 (0.25, 0.31)**	0.38 (0.34, 0.41)**	-0.66 (-0.70, -0.62)**
Social Competence	8.29 (8.27, 8.32)	8.23 (8.20, 8.27)	8.27 (8.24, 8.30)	8.09 (8.06, 8.12)	0.02 (-0.02, 0.06)	-0.03 (-0.08, 0.01)	-0.18 (-0.22, -0.13)**
Emotional Maturity	8.33 (8.32, 8.35)	8.29 (8.27, 8.32)	8.31 (8.29, 8.33)	8.25 (8.22, 8.28)	0.03 (-0.01, 0.06)	-0.02 (-0.05, 0.02)	-0.06 (0.10, -0.02)**
Communication & General Knowledge	7.52 (7.48, 7.55)	7.49 (7.45, 7.53)	7.36 (7.32, 7.40)	7.02 (6.97, 7.06)	0.16 (0.10, 0.21)**	0.13 (0.07, 0.18)**	-0.35 (-0.40, -0.29)**
Physical Health & Well-Being	8.70 (8.69, 8.72)	8.65 (8.63, 8.67)	8.62 (8.60, 8.64)	8.57 (8.55, 8.60)	0.08 (0.06, 0.11)**	0.03 (0.00, 0.06)*	-0.05 (-0.08, -0.02)
	Time Period, Rate (95% CI)				Comparison Δ Rate (95% CI)		
Language & Cognitive Development	0.09 (0.07, 0.11)**	-0.03 (-0.08, 0.02)	-0.41 (-0.44, -0.38)**	0.01 (-0.04, 0.06)	0.51 (0.47, 0.54)**	0.38 (0.33, 0.44)**	0.42 (0.36, 0.48)**
Social Competence	0.04 (0.02, 0.06)**	-0.07 (-0.13, -0.01)*	-0.06 (-0.10, -0.03)**	-0.10 (-0.17, -0.04)*	0.10 (0.06, 0.15)*	0.00 (-0.07, 0.06)	-0.04 (-0.11, 0.03)
Emotional Maturity	0.05 (0.03, 0.07)**	-0.07 (-0.11, -0.02)*	-0.05 (-0.08, -0.02)**	-0.08 (-0.13, -0.03)*	0.11 (-0.07, 0.14)*	0.02 (-0.07, 0.04)	-0.03 (-0.09, 0.03)
Communication & General Knowledge	0.01 (-0.02, 0.04)	-0.09 (-0.16, -0.01)*	-0.13 (-0.18, -0.08)**	-0.08 (-0.16, 0.0)*	0.14 (-0.8, 0.19)**	0.04 (-0.04, 0.13)	0.05 (-0.05, 0.14)
Physical Health & Well-Being	0.01 (-0.00, 0.03)	-0.01 (-0.05, 0.02)	-0.01 (-0.04, 0.01)	-0.01 (-0.05, 0.03)	0.02 (0.00, 0.05)	0.00 (-0.05, 0.04)	0.00 (-0.05, 0.05)

© 2025 Perrigo JL et al. *JAMA Pediatr.*

*p < .05. **p < .001.

© 2025 Perrigo JL et al. *JAMA Pediatr*.

**eTable 2.** Distribution of Missingness: Sex, Ethnoracial Background, and State, 2010-2023 (n = 475 740)	
Variable	Freq. Distribution (%)
Sex	
Male	242,869 (51.1)
Female	232,612 (48.9)
Missing	259 (0.1)
Ethnoracial background	
AA or Black[a]	53,841 (11.3)
AAHPI[b]	34,282 (7.2)
Hispanic or Latino/a	263,037 (55.3)
Other[c]	27,603 (5.8)
White	95,258 (20.0)
Missing	1,719 (0.4)
State	
Arkansas	6,511 (1.4)
California	183,827 (38.6)
Connecticut	4,523 (1.0)
District of Columbia[d]	10,804 (2.3)
Florida	20,032 (4.2)
Illinois	3,300 (0.7)
Kansas	556 (0.1)
Louisiana	5,872 (1.2)
Michigan	7,369 (1.5)
Mississippi	3,072 (0.6)
New York	2,398 (0.5)
North Carolina	1,082 (0.2)
Ohio	656 (0.1)
Oklahoma	7,124 (1.5)
South Carolina	8,957 (1.9)
Tennessee	1,770 (0.4)
Texas	184,596 (38.8)

© 2025 Perrigo JL et al. *JAMA Pediatr*.

Virginia	22,416 (4.7)
Washington	875 (0.2)
Missing	0 (0)

© 2025 Perrigo JL et al. *JAMA Pediatr*.
