# Supplementary material for: COVID-19 Pandemic and the Developmental Health of Kindergarteners
Source: JAMA Pediatr. 2025 Mar 10;179(5):550–8. doi: 10.1001/jamapediatrics.2024.7057 (PMC11894545; doi:10.1001/jamapediatrics.2024.7057)
Supplement: Supplement 2. — Data Sharing Statement [file jamapediatr-e247057-s002.pdf]

Data Sharing Statement

Perrigo. COVID-19 Pandemic and the Developmental Health of Kindergarteners. *JAMA Pediatr*. Published March 10, 2025. doi:10.1001/jamapediatrics.2024.7057

Data
Data available: No
